# Soluble receptor for AGE in diabetic nephropathy and its progression in Finnish individuals with type 1 diabetes

**DOI:** 10.1007/s00125-019-4883-4

**Published:** 2019-05-24

**Authors:** Jenny M. Wadén, Emma H. Dahlström, Nina Elonen, Lena M. Thorn, Johan Wadén, Niina Sandholm, Carol Forsblom, Per-Henrik Groop, on behalf of the FinnDiane Study Group

**Affiliations:** 1 Folkhälsan Research Center, Biomedicum Helsinki, University of Helsinki, Haartmaninkatu 8, 00014 Helsinki, Finland; 2 Abdominal Center Nephrology, University of Helsinki and Helsinki University Hospital, Helsinki, Finland; 3 Research Programs Unit, Diabetes and Obesity, University of Helsinki, Helsinki, Finland; 4 Department of Diabetes, Central Clinical School, Monash University, Melbourne, VIC Australia

**Keywords:** Diabetic nephropathy, End-stage renal disease, Soluble receptor for advanced glycation end-products, Type 1 diabetes

## Abstract

**Aims/hypothesis:**

Activation of the receptor for AGE (RAGE) has been shown to be associated with diabetic nephropathy. The soluble isoform of RAGE (sRAGE) is considered to function as a decoy receptor for RAGE ligands and thereby protects against diabetic complications. A possible association between sRAGE and diabetic nephropathy is still, however, controversial and a more comprehensive analysis of sRAGE with respect to diabetic nephropathy in type 1 diabetes is therefore warranted.

**Methods:**

sRAGE was measured in baseline serum samples from 3647 participants with type 1 diabetes from the nationwide multicentre Finnish Diabetic Nephropathy (FinnDiane) Study. Associations between sRAGE and diabetic nephropathy, as well as sRAGE and diabetic nephropathy progression, were evaluated by regression, competing risks and receiver operating characteristic curve analyses. The non-synonymous SNP rs2070600 (G82S) was used to test causality in the Mendelian randomisation analysis.

**Results:**

Baseline sRAGE concentrations were highest in participants with diabetic nephropathy, compared with participants with a normal AER or those with microalbuminuria. Baseline sRAGE was associated with progression from macroalbuminuria to end-stage renal disease (ESRD) in the competing risks analyses, but this association disappeared when eGFR was entered into the model. The SNP rs2070600 was strongly associated with sRAGE concentrations and with progression from macroalbuminuria to ESRD. However, Mendelian randomisation analysis did not support a causal role for sRAGE in progression to ESRD.

**Conclusions/interpretation:**

sRAGE is associated with progression from macroalbuminuria to ESRD, but does not add predictive value on top of conventional risk factors. Although sRAGE is a biomarker of diabetic nephropathy, in light of the Mendelian randomisation analysis it does not seem to be causally related to progression from macroalbuminuria to ESRD.

**Electronic supplementary material:**

The online version of this article (10.1007/s00125-019-4883-4) contains peer-reviewed but unedited supplementary material, which is available to authorised users.

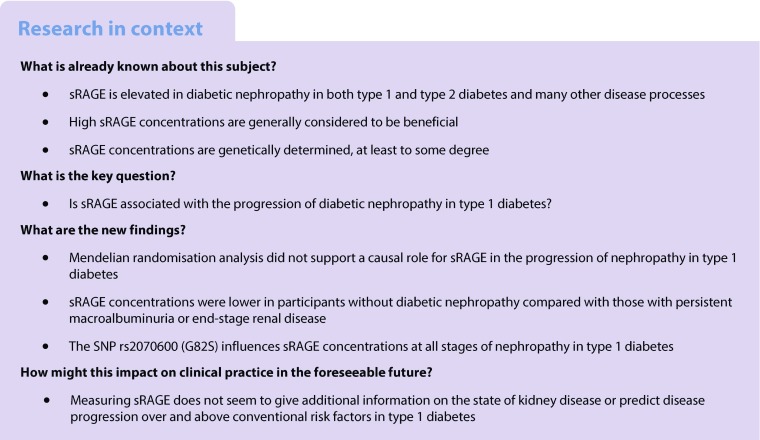



## Introduction

Diabetic nephropathy is associated with increased premature mortality in those with type 1 diabetes [1], but the genetic and biochemical factors involved in the pathogenesis of diabetic nephropathy are not fully understood. The receptor for AGE (RAGE) has been implicated in the development of diabetic nephropathy. RAGE binds several ligands, of which the AGEs are of importance for diabetic complications due to their accelerated formation in the diabetic milieu [2]. The AGE–RAGE interaction initiates deleterious intracellular signalling cascades causing activation of the NF-κB pathway. The soluble isoform of RAGE (sRAGE), which originates from the proteolytic shedding of RAGE by matrix metalloproteinases and to a lesser extent from alternative mRNA splicing [3], works as a decoy receptor by preventing AGEs from binding to the membrane-bound RAGE and thus also prevents RAGE activation. It is therefore hypothesised that sRAGE is a protective factor against the development of diabetic complications. Elevated sRAGE concentrations have been reported in various kidney diseases [4–9] and even in type 1 diabetes [10]. However, no studies on the role of sRAGE in individuals with type 1 diabetes with respect to the presence and progression of diabetic nephropathy have been reported. Therefore, we performed comprehensive analyses on sRAGE in participants with type 1 diabetes with and without diabetic nephropathy.

## Methods

### Participants

A total of 3647 Finnish participants with type 1 diabetes from the nationwide multicentre Finnish Diabetic Nephropathy (FinnDiane) Study were included. Type 1 diabetes was defined as age at onset of 35 years or younger and initiation of insulin treatment within 1 year of diagnosis. Blood and urine samples were collected at baseline and at follow-up (*n* = 3107) for determination of HbA_1c_, AER, serum creatinine and serum sRAGE concentrations (by ELISA; Quantikine, R&D Biosystems, Minneapolis, MN, USA). Other variables, such as clinical events, medication and lifestyle factors, were obtained from a thorough clinical examination and completion of standardised questionnaires by the attending physician. Diabetic nephropathy was defined as AER ≥200 μg/min or ≥300 mg/24 h in two out of three consecutive overnight or 24 h urine collections, and end-stage renal disease (ESRD) as dialysis or kidney transplantation. Normal AER was defined as <20 μg/min or <30 mg/24 h and microalbuminuria as 20–199 μg/min or 30–299 mg/24 h. eGFR was estimated using the Chronic Kidney Disease Epidemiology Collaboration formula [11]. SNP genotyping was performed using the Homogenous Mass Extend MassArray System (Sequenom, San Diego, CA, USA) and the ABI Prism 7900 Sequence Detector System (Applied Biosystems, Foster City, CA, USA) according to the manufacturers’ instructions. The study was approved by the local ethics committees and the study was performed according to the Declaration of Helsinki. All participants provided written informed consent.

### Statistical analyses

For the baseline analyses regarding diabetic nephropathy (yes/no), participants with normal AER and microalbuminuria were compared with participants with macroalbuminuria and ESRD, excluding those with a renal transplant. Log-transformation was used to obtain normal distribution for triacylglycerols and AER. Pearson’s and Spearman’s correlations were applied for univariate correlations. Backward linear regression models were used to evaluate associations between clinical variables and sRAGE concentration, with a *p* value of 0.05 for inclusion of a variable in the model and 0.10 for its removal. Values are given as means ± SD or medians (interquartile range). Analyses were performed using IBM SPSS Statistics 21.0 (IBM Corporation, Somers, NY, USA).

For the analyses related to the progression of kidney disease, Kaplan–Meier and multivariable Cox regression analyses were used, with the logrank *p* value as the measure of significant difference. The Cox model was adjusted for conventional factors that influence the risk of progression of diabetic nephropathy: sex, duration of diabetes, HbA_1c_, BMI, systolic BP, triacylglycerols, AER and eGFR. The competing risks analysis [12] with death as a competing event with progression to ESRD applied a cumulative incidence function and was adjusted for the same variables as the Cox regression. To compare the Cox models with and without sRAGE, receiver operating characteristic (ROC) curve analysis was performed using MedCalc (MedCalc Software, Ostend, Belgium). At the final stage of the assessments of the relationship between sRAGE and ESRD, a Mendelian randomisation analysis was performed with ‘ivregress’ with two-stage least-squares estimator in Stata 11.2 and Stata 15 (Stata Corp LLC, College Station, TX, USA). The Mendelian randomisation approach is comparable with RCTs. In an observational study setting, the genetic markers, which are randomly assigned at meiosis, can be considered the intervention affecting the outcome throughout the lifetime of an individual. ESRD, both prevalent and incident, was used as the endpoint in our analyses and the SNP rs2070600 (G82S) was selected the instrumental variable, as we have previously shown that it strongly influences sRAGE concentrations [13]. A positive association in the Mendelian randomisation analysis would indicate a causal role of sRAGE in the development of ESRD. The SNP’s influence on sRAGE concentrations was estimated using R (R Foundation for Statistical Computing, Vienna, Austria). Power for the Mendelian randomisation was calculated online using mRnd (http://cnsgenomics.com/shiny/mRnd, accessed 19 March 2019) for the binary outcomes.

## Results

### Participant characteristics

Of the total 3647 FinnDiane participants, 2390 (65.5%) had a normal AER, 489 (13.4%) had microalbuminuria, 513 (14.1%) had macroalbuminuria and 255 (7.0%) had ESRD. The clinical characteristics of all participants are summarised in Table 1.

**Table 1:** Participant characteristics at baseline

	Normal AER	Microalbuminuria	Macroalbuminuria	ESRD
*n*	2390	489	513	255
sRAGE, pg/ml	1177 ± 439	1149 ± 472	1588 ± 893	1660 ± 1366
Men, %	48.0	57.9	58.1	59.4
Age, years	35.3 ± 11.9	38.4 ± 12.0	41.1 ± 10.0	45.9 ± 8.2
Type 1 diabetes duration, years	18.6 ± 12.0	25.7 ± 11.1	28.9 ± 8.1	33.6 ± 7.8
AER, mg/24 h	7 (5–12)	56 (26–109)	500 (178–1311)	NA
Serum creatinine, μmol/l	72 (64–81)	77 (68–89)	115 (84–175)	133 (95–363)
eGFR, ml min^−1^ (1.73 m)^−2^	107 (93–122)	101 (82–117)	60 (36–89)	NA
HbA_1c_, %	8.1 ± 1.9	8.8 ± 1.6	8.9 ± 1.9	8.1 ± 2.2
HbA_1c_, mmol/mol	65.2 ± 18.2	72.6 ± 17.2	74.2 ± 19.8	66.3 ± 21.2
BMI, kg/m^2^	24.8 ± 3.4	25.5 ± 3.6	25.9 ± 4.1	24.0 ± 3.7
Systolic BP, mmHg	129 ± 15	136 ± 17	144 ± 20	152 ± 24
Triacylglycerol, mmol/l	0.9 (0.7–1.3)	1.1 (0.8–1.6)	1.4 (1.0–2.0)	1.4 (1.0–1.9)
HDL-cholesterol, mmol/l	1.4 ± 0.4	1.4 ± 0.4	1.2 ± 0.4	1.2 ± 0.4
LDL-cholesterol, mmol/l	2.9 ± 0.8	3.1 ± 0.8	3.4 ± 0.9	3.1 ± 1.0

Data are means ± SD or median (interquartile range), unless otherwise stated

NA, not applicable

The mean ± SD sRAGE concentration in the entire cohort was 1265 ± 656 pg/ml (median 1148 pg/ml, interquartile range 872–1495 pg/ml), with values ranging from 157 to 9621 pg/ml. sRAGE concentrations did not differ between men (1270 ± 676 pg/ml) and women (1259 ± 634 pg/ml). sRAGE concentrations correlated asymptotically with eGFR, meaning that sRAGE concentrations were the highest in individuals with the poorest native kidney function, as we have previously reported [13]. Other clinical variables that correlated with sRAGE concentrations in the univariate analyses in the entire population and separately for kidney-status groups are summarised in Electronic Supplementary Material (ESM) Table 1.

### sRAGE concentrations are associated with albuminuria at baseline

The lowest sRAGE concentrations were seen in participants with a normal AER and in those with microalbuminuria, whereas the highest sRAGE concentrations were observed in participants with macroalbuminuria and ESRD (Table 1). In logistic regression analysis, sRAGE was significantly associated with diabetic nephropathy after correction for confounding factors (sRAGE per 100 pg/ml: OR 1.066, 95% CI 1.020, 1.114; *p* = 0.004). However, the difference in the AUC of the model with (AUC 0.9839) and without sRAGE (AUC 0.9840) was not significant.

In participants with macroalbuminuria, sRAGE was associated with eGFR in linear regression analysis (*β* −0.356, *t* −8.618, *p* = 8.6× 10^−17^). In this group, the lowest sRAGE concentrations (1093 pg/ml) were seen in the highest eGFR quartile (>117 ml min^−1^ [1.73 m]^−2^) and the highest concentrations (1718 pg/ml) in the lowest eGFR quartile (<80.5 ml min^−1^ [1.73 m]^−2^) (*p* = 2.0× 10^−6^).

### Baseline sRAGE concentrations predict progression of diabetic nephropathy in type 1 diabetes but not independently of eGFR

A total of 348 of the 3107 participants with follow-up data available had progressed from a lower to the next stage of kidney disease. The mean ± SD follow-up time was 6.25 ± 2.88 years (range 0–13.6 years).

During follow-up, 147 of 2063 participants developed microalbuminuria but sRAGE was not associated with this change. sRAGE was also not related to progression from micro- to macroalbuminuria (76 progressors among 418 participants). In contrast, every 100 pg/ml increase in sRAGE was associated with a greater risk of progression from macroalbuminuria to ESRD (125 progressors out of 457 participants; HR 1.051, 95% CI 1.040, 1.062, logrank *p* = 3.4 × 10^−20^) in univariate analysis. In multivariable analyses, sRAGE remained a significant predictor of progression to ESRD when sex, duration of type 1 diabetes, HbA_1c_, log triacylglycerols, BMI, systolic BP, history of smoking and log AER were entered into the model (HR 1.027, 95% CI 1.011, 1.043; logrank *p* = 0.001). However, the association disappeared after the addition of eGFR into the model.

Competing risks analyses were performed for progression from macroalbuminuria to ESRD with death as the competing event. The competing risk model was adjusted by the same variables as the Cox regression model. sRAGE showed a clear association with progression to ESRD both as a continuous variable (subdistribution HR 1.000246, 95% CI 1.000022, 1.0047; logrank *p* = 0.032) and as quartiles (subdistribution HR 1.567, 95% CI 1.215, 2.023; logrank *p* = 0.001). However, after entering eGFR into the model, sRAGE and the sRAGE quartile (logrank *p* = 0.058) were no longer significant.

To estimate the value of sRAGE as a predictor of progression, we performed ROC curve analyses on the Cox multivariable model with and without sRAGE. In the ROC curve analysis, the AUC including sRAGE was 0.856 (95% CI 0.816, 0.890) and the AUC without sRAGE was 0.908 (95% CI 0.874, 0.935), with a significant difference in the AUCs (*p* = 0.027).

### SNP and Mendelian randomisation analysis

We have previously reported that sRAGE concentrations are associated with SNPs in the *AGER* gene, with rs2070600 (G82S) having the strongest effect on concentrations [13]. sRAGE concentrations in the different kidney-status groups for the rs2070600 genotype are listed in Table 2. Significant associations were found between sRAGE and this SNP in all kidney-status groups except for participants with ESRD (*p* = 1.2 × 10^−37^ for participants with a normal AER and *p* = 4.2 × 10^−24^ for all participants).

**Table 2:** SNP rs2070600 genotypes and sRAGE concentrations (pg/ml) in the various kidney-status groups

Genotype	Normal AER	*n* (%)	Micro-albuminuria	*n* (%)	Macro-albuminuria	*n* (%)	ESRD	*n* (%)	All participants	*n* (%)
GG	1247 ± 422	1240 (77.9)	1248 ± 483	282 (74.6)	1712 ± 949	341 (77.9)	1709 ± 1428	144 (80.0)	1359 ± 693	2007 (77.6)
GA	960 ± 331	332 (20.9)	908 ± 355	94 (24.9)	1265 ± 638	94 (21.5)	1993 ± 1866	33 (18.3)	1065 ± 653	553 (21.4)
AA	562 ± 297	20 (1.3)	498 ± 249	2 (0.5)	586 ± 402	3 (0.7)	914 ± 371	3 (1.7)	598 ± 315	28 (1.1)
*p* value	1.2 × 10^−37^		1.0 × 10^−9^		1.7 × 10^−5^		NS		4.2 × 10^−24^	

Data are means ± SD unless otherwise indicated

The *p* values indicate the influence of the SNP genotype on the sRAGE concentrations within the groups, calculated by one-way ANOVA

NS, not significant

The SNP rs2070600 was associated with progression from macroalbuminuria to ESRD in the univariate model (HR 1.13, 95% CI 1.07, 1.19; logrank *p* = 1.2 × 10^−5^) as well as in a multivariable model adjusted for sex, duration of diabetes, HbA_1c_, log triacylglycerols, BMI, SBP and history of smoking (HR 1.08, 95% CI 1.02, 1.14; logrank *p* = 0.01). However, after entering log AER and eGFR into the model, the association between rs2070600 and progression from microalbuminuria to ESRD was no longer significant.

In order to evaluate whether sRAGE plays a causative role in progression from macroalbuminuria to ESRD, we performed a Mendelian randomisation analysis. This methodology mimics an RCT in such a way that the alleles assigned randomly at meiosis are unbiased by other confounding factors influencing the outcome; in this case, progression to ESRD. The SNP rs2070600 was used as the instrumental variable, and explains about 4.2% of the sRAGE variation in our population. sRAGE was used as the endogenous variable and ESRD as the main outcome. This analysis provided no evidence for a causative role of sRAGE in the development of ESRD (OR −1.89 × 10^−5^, 95% CI −1.10 × 10^−4^, 7.24 × 10^5^; *p* = 0.685) with an estimated power of 92%.

## Discussion

This study explored the potential role of sRAGE in the development of diabetic nephropathy and particularly a causal role in progression from macroalbuminuria to ESRD. Our data showed that sRAGE is associated with diabetic nephropathy at baseline, and that baseline sRAGE is also associated with progression from macroalbuminuria to ESRD. However, further analyses showed that sRAGE does not add any predictive value on top of other conventional risk factors for diabetic nephropathy and that sRAGE is not causally related to progression from macroalbuminuria to ESRD.

High sRAGE concentrations have previously been observed in individuals with poor kidney function, albeit with different underlying causes [4], and those with different types of diabetes [5], type 1 diabetes [10] and type 2 diabetes [6, 7], and also in individuals without diabetes [8, 9]. It is noteworthy that our results are in line with these previous findings, as sRAGE concentrations were the highest in participants with diabetic nephropathy at baseline.

There is a strong inverse correlation between sRAGE and eGFR in individuals with type 1 [10, 13] and type 2 diabetes [14], and even in individuals with non-diabetic chronic kidney disease [9, 15]. The strong inverse correlation between sRAGE and eGFR in participants with macroalbuminuria observed in the present study might explain why sRAGE was no longer significantly associated with progression to ESRD in the Cox or competing risk regression analyses. Notably, also in the macroalbuminuric group, sRAGE concentrations were low when eGFR was normal and high when eGFR was low, suggesting that eGFR strongly influences sRAGE concentrations. Whether it is the amount of sRAGE filtered into the urine that is affected by the decline in eGFR or that the formation of sRAGE is increased because of reduced kidney function is not known. However, it has been suggested that kidney failure directly influences circulating sRAGE concentrations [15].

To evaluate the potential role of sRAGE as an additional risk factor for progression from macroalbuminuria to ESRD, we performed a ROC curve analysis. In the ROC curve analysis, sRAGE did not improve the AUC when entered into the model. This suggests that sRAGE does not, at least in our population, add new predictive value on top of conventional risk factors for the development of ESRD.

We furthermore performed a Mendelian randomisation analysis in a well-powered population to evaluate whether sRAGE is causally involved in the process leading to ESRD. This analysis indicated that sRAGE is not causally related to ESRD, at least in our participants with type 1 diabetes. The Mendelian randomisation method is not uncomplicated and pleiotropy, for example, might influence and bias the outcome, especially if causality is observed. In our case, the Mendelian randomisation results did not support a causal relationship between sRAGE and ESRD, and the risk of our results being biased using this method is small. Taken together, both the conventional and other statistical methods suggest that although sRAGE is associated with worsening kidney function, it is not causally involved in the disease process.

Notably, the present study was observational and not a mechanistic study, and therefore we can only speculate about the underlying mechanisms that increase sRAGE concentration in the presence of kidney disease. As already pointed out, sRAGE does not seem to be causally involved in disease progression in light of our results, but it is nevertheless possible that the cell-membrane-bound RAGE plays an important role in the pathogenesis of diabetic nephropathy through its ability to activate intracellular pathways. RAGE activation through ligand binding initiates deleterious intracellular pathways leading, for example, to increased expression of cyto- and chemokines and other inflammatory genes, causing a chronic inflammatory state [16, 17]. If the association we observed between the SNP rs2070600 (G82S) and progression to ESRD is a true positive finding then this might reflect the different degrees of RAGE activation caused by the different SNP alleles [18, 19] and a more pronounced inflammatory environment perhaps predisposing to ESRD development. The 82 serine residue of the SNP rs2070600 is known to enhance binding of the S100A12 ligand and AGEs. This results in increased NF-κB expression [19, 20] and also enhanced *N*-linked glycosylation at Asn81, which influences the ligand-binding abilities of RAGE [21]. These differences might translate into changes in the function of RAGE, but not necessarily into the function of sRAGE. Therefore, suggestions that sRAGE is a marker of RAGE activation, rather than being involved in the disease processes [13, 22], might hold true.

As high sRAGE concentrations are generally considered beneficial in other conditions [23, 24], it is unclear why the high sRAGE concentrations associated with the decline in renal function are not advantageous and why sRAGE seems to behave more like a risk factor rather than the protective factor it is considered to be. Notably, sRAGE does not, to current knowledge, convey deleterious effects when ligands are bound and thereby prevents ligands from interacting with RAGE [17, 25]. In non-diabetic chronic kidney disease, high sRAGE is associated with smaller intima–media thickness [26], and a low concentration of endogenous sRAGE is negatively correlated with the aortic calcification index [27]. In individuals with and without diabetes undergoing peritoneal dialysis, a low sRAGE concentration has been associated with accelerated atherosclerosis [28], and in individuals with and without diabetes undergoing haemodialysis, the vascular calcification index has been reported to be lower in those with higher sRAGE concentrations [29]. Taken together, these findings suggest that sRAGE may in some circumstances be considered beneficial in kidney disease. Such a view is further supported by animal studies in which administration of sRAGE was associated with a reduced level or amelioration of atherosclerotic plaques [30, 31]. It could even be speculated that sRAGE is not a marker of chronic kidney disease, but rather of atherosclerosis or chronic inflammation. However, this requires further study. In type 1 diabetes, we and others have shown that sRAGE is associated with cardiovascular mortality independently of kidney disease [10, 13]. It could also be that sRAGE in our participants with macroalbuminuria is, through unknown mechanisms, biologically inactive and accumulates in the circulation because of a decrease in the eGFR and therefore loses its beneficial effects. Another possibility is that sRAGE concentrations in renal disease are not high enough to perform an efficient scavenger function [25, 32] and this phenomenon might be even more prominent in diabetes, in which large amounts of RAGE ligands such as AGEs are present. This notion might lead to our results being interpreted as indicating that sRAGE is not beneficial in renal disease, as high concentrations are related to worse outcomes. It has recently been proposed that sRAGE alone may not be a sufficiently good marker and that the AGE:sRAGE ratio may be more informative in diseases such as diabetes and renal disease in predicting the disease process [32, 33]. In future studies, the ratio between AGEs and sRAGE could be more thoroughly explored in participants with type 1 diabetes and kidney disease in order to clarify the discrepancy between a high sRAGE concentration and disease outcome. Thus, it is also possible that interventions that leads to a further elevation of sRAGE concentrations could have therapeutic effects in individuals with diabetes and those with kidney disease.

To summarise, we have shown that sRAGE is associated with diabetic nephropathy and its progression in individuals with type 1 diabetes, but also that sRAGE is a mere marker of diabetic nephropathy and not necessarily related to the disease process itself. However, these data do not exclude the possibility that membrane-bound RAGE is involved in the pathogenesis of diabetic nephropathy.

## Electronic supplementary material

ESM Table 1 (PDF 169 kb)

## Data Availability

The datasets generated during and/or analysed during the current study are available from the corresponding author on reasonable request.

## Abbreviations

ESRD: End-stage renal disease
RAGE: Receptor for AGE
ROC: Receiver operating characteristic
sRAGE: Soluble receptor for AGE
