# Supplementary material for: Soluble receptor for AGE in diabetic nephropathy and its progression in Finnish individuals with type 1 diabetes
Source: Diabetologia. 2019 May 24;62(7):1268–74. doi: 10.1007/s00125-019-4883-4 (PMC6559996; doi:10.1007/s00125-019-4883-4)
Supplement: Supplementary file 1 — (PDF 169 kb) [file 125_2019_4883_MOESM1_ESM.pdf]

Supplementary Table 1. Univariate correlations between baseline sRAGE concentrations and clinical variables in different kidney status groups. Only the significant correlations are shown with numerals. For AER, eGFR, serum creatinine, and triacylglycerol Spearman's correlation coefficients are shown.

Variable	Normo	Micro	Macro	ESRD	All patients
Age (years)	-0.74*	-0.096	-	-	0.034
Duration (years)***	-0.102**	-	-	-	0.045
AER(mg/24-h)***	-	-	0.117	-	0.058*
eGFR(ml/min/1.73m^2)*, ***	-	-	-0.385**	-0.583**	-0.157**
s-creatinine (µmol/l)***	-	-	0.388**	0.527**	0.192**
HbA_{1c} (%) ***	-	-	-	-	0.045
Total cholesterol (mmol/l)	-	-	-	-	-
LDL cholesterol (mmol/l)***	-	-	-	-	0.049
HDL cholesterol (mmol/l)***	-	-	-	-0.232*	-0.099**
Triacylglycerol (mmol/l)***	-	-	-	-	0.035
BMI (kg/m^2) ***	-0.169**	-0.213**	-0.202**	-	-0.135**
WHR, men	-0.215**	-0.220	-	-	-
WHR, women	-0.116*	-0.127	-	-	-
SBP (mmHg)	-0.120**	-	0.143*	-	0.077**
DBP (mmHg)	-0.108**	-	-	-	-
MAP (mmHg)	-0.132**	-	0.133	-	0.057*
PP (mmHg)	-0.065	-	0.105	-	0.073**

Variables entered in the initial multivariable linear regression: log AER, age, BMI, eGFR, duration of diabetes, HbA1c, HDL, LDL, systolic and diastolic blood pressure, mean arterial pressure, pulse pressure, serum creatinine, log triacylglycerol

*$p \leq 0.001$

**$p \leq 0.00001$

*** Variables significantly associated with sRAGE in the linear regression model including all patients

As sRAGE concentrations are influenced by the kidney status we also analysed the groups separately to establish correlations between sRAGE concentrations and clinical variables. We used multivariable linear regression models to establish which clinical factors were independently associated with sRAGE concentrations in the entire cohort (table 1).
